# Supplementary material for: A Narrative Review of Prognostic Gene Signatures in Oral Squamous Cell Carcinoma Using LASSO Cox Regression
Source: Biomedicines. 2025 Jan 8;13(1):134. doi: 10.3390/biomedicines13010134 (PMC11759772; doi:10.3390/biomedicines13010134)
Supplement: Supplementary file 1 [file biomedicines-13-00134-s001.zip › Supplementary Table 1 and 2.pdf]

Supplementary table 1. A brief presentation of statistical tools (considering pros and cons) used in survival prediction in cancer research.

Statistical Tool	Description	Pros	Cons
**Cox Proportional Hazards**	Describes the risk of an event based on various covariates.	- Comprehensible and transparent outcomes (hazard ratios).	- Faces challenges when dealing with high-dimensional datasets.
		- Appropriate for small to moderately sized datasets.	- - There is a danger of overfitting when using an excessive number of predictors.
**Random Survival Forests (RSF)**	An ensemble learning technique that adapts random forests for survival analysis.	- Manages relationships that are non-linear and accounts for interactions between predictors.	-Results can be harder to interpret.
		- Resilient against outliers and issues related to multicollinearity.	- Analyzing large datasets can be resource-intensive.
**Elastic Net Regression**	Integrates L1 (lasso) and L2 (ridge) penalties for the purposes of feature selection and regularization.	-Effectively deals with multicollinearity issues.	- It demands more computational resources compared to LASSO.
		- Appropriate for situations with correlated predictors.	- Careful adjustment of hyperparameters is necessary.
**LASSO Cox Regression**	A penalized regression approach that merges feature selection with regularization.	- Streamlines models by reducing insignificant coefficients to zero	- The choice of hyperparameters (penalty factor λ) is crucial.
		Well-suited for datasets with a high number of dimensions.	- It might overlook weak but significant predictors.
		- Improves the clarity of interpretation.	

Supplementary table 2. Justification of using LASSO Cox Regression in cancer research.

Criteria	Justification
**Suitability for High-Dimensional Data**	Processes a vast number of genes, perfect for genomic datasets related to OSCC.
**Improved Interpretability**	Reduces insignificant coefficients to zero, keeping only the most important predictors.
**Robustness in Model Creation**	Prevents overfitting through the use of a shrinkage method.
**Proven Effectiveness**	Confirmed in 34 studies for forecasting patient outcomes through independent prognostic gene signatures.
**Actionable Insights**	Discovers genetic markers associated with essential cancer pathways, supporting progress in treatments.
