# Supplementary material for: A Narrative Review of Prognostic Gene Signatures in Oral Squamous Cell Carcinoma Using LASSO Cox Regression
Source: Biomedicines. 2025 Jan 8;13(1):134. doi: 10.3390/biomedicines13010134 (PMC11759772; doi:10.3390/biomedicines13010134)
Supplement: Supplementary file 1 [file biomedicines-13-00134-s001.zip › Supplementary table 3.pdf]

Supplementary table 3. List of FDA-Approved Drugs in OSCC

FDA-approved drugs	Commercial name	Producer	Year of approval	Pharmaceutical formulation	Dose	Mechanism Of Action
L-cysteine	Elcys	Exela Pharma Sciences, LLC	2019	Injection, solution	10 mg/1mL	Works by donating a thiol group to facilitate glutathione synthesis, thereby reducing oxidative stress and promoting cellular redox balance.
Bimatoprost	Durysta	Allergan, Inc.	2020	Implant	10 ug/1	Works by increasing the outflow of aqueous humor through the uveoscleral pathway, reducing intraocular pressure in conditions like glaucoma.
Diclofenac	Cambia	Nautilus Neurosciences, Inc.	2010	Powder, for solution	1 mg/1mg	Works by inhibiting cyclooxygenase (COX) enzymes, reducing the production of prostaglandins, thereby alleviating pain and inflammation.
Rutin	N/A	N/A	N/A	N/A	N/A	A flavonoid that acts as an antioxidant, reducing oxidative stress and supporting blood vessel health by strengthening capillaries and improving circulation.
L-glutamic acid	N/A	N/A	N/A	N/A	N/A	An excitatory neurotransmitter in the brain, promoting neuronal communication and playing a key role in learning and memory.
Ephedrine-racemic	N/A	N/A	N/A	N/A	N/A	A mixture of two enantiomers that acts as a sympathomimetic, stimulating alpha and beta-adrenergic receptors to increase heart rate, blood pressure, and bronchodilation.
PGL5001 (Bentamapimod)	N/A	N/A	N/A	N/A	N/A	A selective inhibitor of p38 MAP kinase, which reduces inflammation and modulates immune responses, potentially helping in the treatment of autoimmune diseases and inflammatory conditions.
RGB-286638	N/A	N/A	N/A	N/A	N/A	A kinase inhibitor targeting CLK2, involved in regulating cell cycle and splicing.
Fenobibric-acid	N/A	N/A	N/A	N/A	N/A	Activates peroxisome proliferator-activated receptors (PPARs), helping to reduce cholesterol and triglyceride levels, and improve lipid profiles in the treatment of dyslipidemia.

Nifllumic acid	N/A	N/A	N/A	N/A	N/A	A nonsteroidal anti-inflammatory drug (NSAID) that inhibits cyclooxygenase (COX).
Coumarin	N/A	N/A	N/A	N/A	N/A	Acts as an anticoagulant by inhibiting vitamin K epoxide reductase, affecting clotting factor synthesis.
Curcumin	Victory 19 Virus Out	CHANGJAE BENKO BIO Co., Ltd.	2022	Liquid	0.5 g/1mL	Inactivates viruses by disrupting their lipid membranes, denaturing proteins, and using oxidative agents like hydrogen peroxide to damage viral components.
Ellagic-acid	N/A	N/A	N/A	N/A	N/A	Ellagic acid exerts its effects by scavenging free radicals, reducing inflammation, and inhibiting cancer cell growth through modulation of cellular pathways.
Ethoxzolamide	N/A	N/A	N/A	N/A	N/A	Inhibits carbonic anhydrase, reducing bicarbonate production and lowering intraocular pressure, used in glaucoma treatment.
Ferulic-acid	N/A	N/A	N/A	N/A	N/A	An antioxidant that neutralizes free radicals, protects against oxidative stress, and has anti-inflammatory and anti-cancer properties.
Hydrochlorothiazide	Hydrochlorothiazide	Sanis Health Inc	2011	Tablet	25 mg	A diuretic that inhibits sodium reabsorption in the kidneys, leading to increased urine output and reduced blood pressure.
Indisulam	N/A	N/A	N/A	N/A	N/A	An anticancer agent that inhibits the function of the enzyme p53 and induces cell cycle arrest and apoptosis in cancer cells.
Mafenide	Sulfamylon	Rising Pharma Holdings, Inc.	2020	Topical	85 mg/1g	A topical antibiotic that inhibits bacterial folic acid synthesis, preventing bacterial growth and infection in burn wounds.
Para-toluenesulfonamide	N/A	N/A	N/A	N/A	N/A	Inhibits bacterial folic acid synthesis, preventing bacterial growth.
Saccharin	N/A	N/A	N/A	N/A	N/A	N/A
U-104	N/A	N/A	N/A	N/A	N/A	A carbonic anhydrase inhibitor that works by reducing the production of bicarbonate ions, thereby decreasing fluid secretion and lowering intraocular pressure.

Zonisamide	Zonegran	Advanz Pharma (US) Corp.	2000	Capsule	25 mg/1	An anticonvulsant that works by inhibiting sodium and calcium channels, stabilizing neuronal activity, and preventing seizures.
Capecitabine	Capecitabine	Sanis Health Inc	2021	Tablet	500 mg	An oral chemotherapy drug that is converted into 5-fluorouracil (5-FU) in the body, inhibiting DNA synthesis and interfering with cancer cell growth.
Carmofur	N/A	N/A	N/A	N/A	N/A	An antineoplastic agent that inhibits thymidylate synthase, disrupting DNA synthesis and preventing the growth of cancer cells.
Doxifluridine	N/A	N/A	N/A	N/A	N/A	A chemotherapy drug that is converted into 5-fluorouracil (5-FU) in the body, inhibiting thymidylate synthase and disrupting DNA synthesis in cancer cells.
Floxuridine	Floxuridine	JND Therapeutics, Inc.	2022	Injection, powder, lyophilized, for solution	500 mg/1	Floxuridine is converted to 5-FU, inhibiting thymidylate synthase and disrupting DNA synthesis in cancer cells.
Enocitabine	N/A	N/A	N/A	N/A	N/A	A nucleoside analog that inhibits DNA synthesis by incorporating into the DNA chain and blocking further elongation, ultimately leading to cell death in cancer cells.
Ftorafur	N/A	N/A	N/A	N/A	N/A	A chemotherapy drug that is converted into 5-fluorouracil (5-FU) in the body, inhibiting thymidylate synthase and disrupting DNA synthesis in cancer cells.
Gemcitabine	Gemcitabine	Accord Healthcare, S.L.U.	2018	Injection, solution	100 mg/1mL	A nucleoside analog that inhibits DNA synthesis by incorporating into the DNA chain and inhibiting ribonucleotide reductase, leading to cell cycle arrest and cell death in cancer cells.
Gemcitabine elaidate	N/A	N/A	N/A	N/A	N/A	A prodrug of gemcitabine, which is metabolized into gemcitabine in the body to inhibit DNA synthesis and promote cancer cell death.
Nolatrexed	N/A	N/A	N/A	N/A	N/A	An antifolate drug that inhibits thymidylate synthase, leading to a reduction in DNA synthesis and the growth of cancer cells.
Pemetrexed	Alimta	Eli Lilly Nederland B.V.	2016	Injection, powder, for solution	500 mg	An antifolate chemotherapy drug that inhibits enzymes involved in folate metabolism, disrupting DNA and RNA synthesis in cancer cells.

Raltitrexed	Tomudex	Pfizer Italia S.R.L.	1996	Powder, for solution	2 mg / vial	An antifolate chemotherapy drug that inhibits thymidylate synthase, disrupting DNA synthesis and inhibiting cancer cell growth.
Trifluridine	Viroptic	Bausch & Lomb Incorporated	1987	Solution	1 %	An antiviral medication that inhibits DNA synthesis by incorporating into viral DNA, disrupting replication and preventing the spread of herpes simplex virus.
Trimethoprim	PrimSol	Allegis Holdings, Llc	2017	Solution	50 mg/5mL	An anticonvulsant that works by increasing GABA activity in the brain, helping to prevent seizures.
5-fluorouracil	Fluorouracil	Alembic Pharmaceuticals Limited	2023	Injection, solution	50 mg/1g	Inhibits thymidylate synthase, disrupting DNA synthesis and preventing the growth of cancer cells.
5-FP	N/A	N/A	N/A	N/A	N/A	Analog of proline that inhibits protein synthesis and disrupts cell function by interfering with protein translation and amino acid metabolism in cancer cells.
Pidolic-acid	N/A	N/A	N/A	N/A	N/A	Acts as an antioxidant, supporting amino acid metabolism and protecting cells from oxidative stress.
Trometamol	Tham	Hospira, Inc.	2020	Injection, solution	3.6 g/100mL	A buffering agent that helps regulate pH by neutralizing excess acid in the blood, commonly used in the treatment of metabolic acidosis.
Vandetanib	Caprelsa	Genzyme Corporation	2022	Tablet, film coated	300 mg/1	Inhibits tyrosine kinases, blocking cancer cell growth and angiogenesis in medullary thyroid cancer.
L-citrulline	N/A	N/A	N/A	N/A	N/A	L-citrulline is converted into L-arginine in the body, which then increases the production of nitric oxide, leading to vasodilation and improved blood flow.
Amiloride	Amiloride Hydrochloride	Physicians Total Care, Inc.	2005	Tablet	5 mg/1	A potassium-sparing diuretic that works by inhibiting sodium channels in the kidneys, reducing sodium reabsorption and increasing urine output without causing potassium loss.

Mexiletine	Mexiletine HCl	Watson Pharmaceuticals	2008	Capsule	200 mg/1	A sodium channel blocker that stabilizes cardiac cell membranes, preventing abnormal electrical activity and treating ventricular arrhythmias.
Aloxistatin	N/A	N/A	N/A	N/A	N/A	A protease inhibitor that blocks the activity of serine proteases, preventing the breakdown of proteins and inhibiting tumor growth, often investigated for its anticancer effects.
Delanzomib	N/A	N/A	N/A	N/A	N/A	A proteasome inhibitor that blocks the degradation of proteins by the proteasome, leading to the accumulation of damaged proteins and inducing cell death in cancer cells.
AZD4282	N/A	N/A	N/A	N/A	N/A	Inhibits the P2X7 receptor, modulating immune response and inflammation.
MC-1	N/A	N/A	N/A	N/A	N/A	A melanocortin receptor 1 agonist that stimulates melanin production, potentially used for skin protection and photoprotection.
Mimosin	N/A	N/A	N/A	N/A	N/A	A tyrosine analog that inhibits ribonucleotide reductase, disrupting DNA synthesis and cell division.
Azathioprine	Imuran	Aspen Pharmacare Canada Inc.	2001	Powder, for solution	50 mg / vial	An immunosuppressive drug that inhibits DNA synthesis, blocking T-cell and B-cell proliferation to prevent organ rejection and treat autoimmune diseases.
C11-Acetate	N/A	N/A	N/A	N/A	N/A	C11-acetate is used in PET imaging to assess metabolic activity by highlighting areas of increased tissue uptake.
Mercaptopurine	Purinethol	Stason Pharmaceuticals, Inc.	2022	Tablet	50 mg/1	An antimetabolite that inhibits DNA synthesis by interfering with purine metabolism.
Polyiosine	N/A	N/A	N/A	N/A	N/A	An antiviral agent that inhibits viral replication by interfering with viral RNA synthesis.

Cladribine	Cladribine Injection	Generic Medical Partners Inc	2020	Solution	1 mg / mL	Purine analog that inhibits DNA synthesis, leading to cell death, particularly in lymphocytes.
ABT-702	N/A	N/A	N/A	N/A	N/A	A selective inhibitor of adenosine deaminase that enhances adenosine signaling, potentially used for immune modulation and treating autoimmune diseases.
Adenosine-phosphate	N/A	N/A	N/A	N/A	N/A	Acts as a precursor in ATP production, providing energy for cellular processes and regulating cellular signaling pathways.
Ribavirin	Ibavyr	Pendopharm Division Of Pharmascience Inc	2014	Tablet	400 mg	An antiviral drug that inhibits RNA synthesis by interfering with viral RNA-dependent RNA polymerase.
L-aspartic-acid	N/A	N/A	N/A	N/A	N/A	An amino acid that plays a role in protein synthesis and neurotransmission, supporting cellular energy production and metabolic pathways.
Glutathione	K2LTS Brain Rhabilitation	K2LT LAB, LLC	2024	Liquid	23 g/1001	Promotes cognitive recovery and neuroplasticity to enhance brain function.
Cladribine	Cladribine Injection	Fresenius Kabi Italia S.R.L.	2009	Solution	1 mg / mL	A purine analog that inhibits DNA synthesis by incorporating into DNA and disrupting replication, leading to cell death
Dipyridamole	Dipyridamole	Hospira, Inc.	2006	Injection, solution	5 mg/1mL	Inhibits platelet aggregation and causes vasodilation
Fludarabine	Fludara	Sanofi Aventis Deutschl	2002	Tablet	10 mg	An antimetabolite that inhibits DNA synthesis by interfering with purine metabolism, leading to cell death

		and GmbH				
Pentostatin	Nipent	Hospira, Inc.	2007	Injection, powder, lyophilized, for solution	2 mg/1mL	Inhibits adenosine deaminase, disrupting purine metabolism and causing cancer cell death.
Vidarabine	N/A	N/A	N/A	N/A	N/A	Inhibits viral DNA polymerase, preventing viral DNA synthesis and replication
Pidolic-acid	N/A	N/A	N/A	N/A	N/A	Acts as an antioxidant, scavenging free radicals and helping to reduce oxidative stress and support cellular function.
KY02111	N/A	N/A	N/A	N/A	N/A	Modulates S1P receptors, sequestering immune cells in lymph nodes and reducing autoimmune activity.
WAY-316606	N/A	N/A	N/A	N/A	N/A	A selective inhibitor of Nedd8-activating enzyme (NAE), blocking the neddylation process, which is involved in the regulation of the ubiquitin-proteasome system
Polyinosine	N/A	N/A	N/A	N/A	N/A	Stimulates RNA synthesis and enhances the immune response, aiding in viral inhibition and immune activation.
LMI070	N/A	N/A	N/A	N/A	N/A	Modulates RNA splicing to correct SMN2 gene splicing, increasing SMN protein production in Spinal Muscular Atrophy (SMA) patients.
A-1120	N/A	N/A	N/A	N/A	N/A	A small molecule inhibitor of NAD+ biosynthesis, specifically targeting the NAD+ synthetase (NMNAT) pathway.
Naringenininc-acid	N/A	N/A	N/A	N/A	N/A	N/A
L-proline	N/A	N/A	N/A	N/A	N/A	An amino acid that plays a key role in collagen synthesis and protein metabolism, supporting tissue repair and wound healing.
Succinic-acid	N/A	N/A	N/A	N/A	N/A	A metabolic intermediate in the Krebs cycle, enhancing ATP production and supporting cellular energy metabolism.
Oseltamivir-phosphate	Ebilfumin	Actavis Group Ptc Ehf.	2016	Capsule	45 mg	Modulate immune responses and exhibit anti-inflammatory and antiviral effects, potentially by influencing immune cell activity and cytokine production.
Acetazolamide	Acetazolamide	Aa Pharma Inc	1982	Tablet	250 mg	Inhibits carbonic anhydrase, reducing bicarbonate reabsorption, increasing urine output, and lowering intraocular pressure.

JTE-607	N/A	N/A	N/A	N/A	N/A	Inhibits Mcl-1, promoting apoptosis in cancer cells.
Amiloride	Amiloride Hydrochloride	Physicians Total Care, Inc.	2005	Tablet	5 mg/1	Inhibits sodium channels in the kidneys, reducing sodium reabsorption and increasing urine output without causing potassium loss.
BC-11	N/A	N/A	N/A	N/A	N/A	N/A
Mexiletine	Mexiletine HCl	Watson Pharmaceuticals	2008	Capsule	200 mg/1	A sodium channel blocker that stabilizes the cardiac cell membrane, reducing abnormal electrical activity.
4-chlorophenylguanidine	N/A	N/A	N/A	N/A	N/A	A selective agonist of the 5-HT3 receptor (serotonin receptor), which can modulate serotonergic signaling in the brain
2-deoxyglucose	N/A	N/A	N/A	N/A	N/A	Inhibits glycolysis, disrupting glucose metabolism and depleting cellular energy.
Benzoic acid	N/A	N/A	N/A	N/A	N/A	Acts as an antimicrobial agent by disrupting cell membranes.
Diminazene-aceturate	N/A	N/A	N/A	N/A	N/A	Inhibits DNA and RNA synthesis in protozoa.
Ceritinib	Zykadia	Novartis Farma S.P.A.	2019	Tablet, film coated	150 mg/1	A tyrosine kinase inhibitor that targets anaplastic lymphoma kinase (ALK), inhibiting its activity to block tumor cell growth
Gilteritinib	Xospata	Astellas Pharma Europe Bv	2018	Tablet	40 mg/1	A tyrosine kinase inhibitor that targets FLT3 mutations (including internal tandem duplications and point mutations), inhibiting FLT3 signaling.
Midostaurin	Rydapt	Novartis Farma S.P.A.	2017	Capsule, liquid filled	25 mg/1	A kinase inhibitor that targets FLT3, KIT, and other kinases, blocking signaling pathways involved in cancer cell growth,
3-indolebutyric-acid	N/A	N/A	N/A	N/A	N/A	N/A

Fluoxetine	Act Fluoxetine	Dr. Reddy's Laboratories Limited	N/A	Capsule	40 mg	Inhibits the reuptake of serotonin, increasing its levels in the brain.
Tannic acid	N/A	N/A	N/A	N/A	N/A	Works by binding to proteins and other macromolecules, exerting antioxidant, anti-inflammatory, and antimicrobial effects.
Adenosine-triphosphate	N/A	N/A	N/A	N/A	N/A	Primary energy carrier in cells, facilitating cellular processes like muscle contraction, protein synthesis, and nerve transmission by transferring energy through phosphate group hydrolysis
Alvocidib	N/A	N/A	N/A	N/A	N/A	Inhibits cyclin-dependent kinases (CDKs), causing cell cycle arrest and promoting apoptosis in cancer cells.
AT-7519	N/A	N/A	N/A	N/A	N/A	Inhibits cyclin-dependent kinases (CDKs), leading to cell cycle arrest and apoptosis in cancer cells.
Dinaciclib	N/A	N/A	N/A	N/A	N/A	Inhibits cyclin-dependent kinases (CDKs), leading to cell cycle arrest and apoptosis in cancer cells.
Indirubin	N/A	N/A	N/A	N/A	N/A	Inhibits CDKs and GSK-3β, leading to cell cycle arrest and apoptosis in cancer cells.
PHA-793887	N/A	N/A	N/A	N/A	N/A	CDK7 inhibitor that blocks CDK7 activity, leading to cell cycle arrest and apoptosis in cancer cells.
Dehydroepiandrosterone	Intrarosa	Millicent US, Inc.	2020	Insert	6.5 mg/1	Acts as a precursor hormone for androgens and estrogens, influencing various physiological processes including immune modulation, mood regulation, and bone health
RRx-001	N/A	N/A	N/A	N/A	N/A	Acts as a histone deacetylase (HDAC) inhibitor and epigenetic modulator, enhancing chemotherapy efficacy, reversing tumor resistance, and modulating the immune response
Chloramphenicol	Chloromycetin	Parke Davis Spa	2006	Ointment	10 mg/1g	Inhibits protein synthesis by binding to the 50S ribosomal subunit, preventing peptide bond formation in bacteria.
