# Supplementary material for: A Narrative Review of Prognostic Gene Signatures in Oral Squamous Cell Carcinoma Using LASSO Cox Regression
Source: Biomedicines. 2025 Jan 8;13(1):134. doi: 10.3390/biomedicines13010134 (PMC11759772; doi:10.3390/biomedicines13010134)
Supplement: Supplementary file 1 [file biomedicines-13-00134-s001.zip › Supplementary table 4.pdf]

Supplementary table 4. List of abbreviations and the full form and definitions.

Abbreviations	Full form	Definition
OSCC	Oral squamous cell carcinoma	Oral squamous cell carcinoma is a type of cancer that originates from the squamous cells lining the inside of the mouth, including the tongue, gums, cheeks, and floor of the mouth, and is the most common form of oral cancer.
HNSCC	Head and neck squamous cell carcinoma	a type of cancer that originates in the squamous cells lining the mucosal surfaces of the head and neck region, including the mouth, throat, and nasal cavity, essentially meaning it is a cancer that develops from the flat, thin cells that line these areas; it is the most common type of head and neck cancer, often associated with tobacco use and heavy alcohol consumption
TCGA	Cancer Genome Atlas	The Cancer Genome Atlas (TCGA) is a landmark cancer genomics program that sequenced and molecularly characterized over 11000 cases of primary cancer samples.
LASSO	least absolute shrinkage and selection operator	a regression analysis method that performs variable selection and regularization to improve the accuracy and interpretability of a statistical model
Tregs	Regulatory T cell	A type of white blood cell that regulate the immune system and prevent autoimmune disease
FDA	Food and Drug Administration	The Food and Drug Administration (FDA) is responsible for protecting the public health by assuring the safety, efficacy, and security of human and veterinary drugs, biological products, medical devices, our nation's food supply, cosmetics, and products that emit radiation.
HPV	Human papillomavirus	a group of viruses that can cause genital warts, cancers, and other health problems
TP53	tumor protein p53,	is a gene that produces a protein that controls cell division and cell death.
CDKN2A	cyclin-dependent kinase inhibitor 2A	a tumor suppressor gene that produces proteins that prevent cells from growing and dividing too quickly or in an uncontrolled way.
SCC	Squamous Cell Carcinoma	Squamous Cell Carcinoma is a malignant tumor that arises from squamous cells.
USP9X	Ubiquitin Specific Peptidase 9, X-linked.	a gene that encodes a protein involved in controlling protein degradation and stability by removing ubiquitin molecules. It plays a role in cell survival, invasion, and migration in various types of cancer.
MLL4	Mixed-Lineage Leukemia 4	a gene that encodes a protein involved in the regulation of gene expression during development. It plays a role in histone modification and chromatin remodeling. Mutations in this gene are linked to certain cancers.

ARID2	AT-rich Interactive Domain 2	a gene that encodes a protein involved in chromatin remodeling. It is part of the ARID family, which regulates gene expression by influencing DNA structure. Mutations are associated with various cancers.
UNCBC	Uncommon Clusters of Base Composition	It is a term used in bioinformatics to refer to unusual patterns in DNA base compositions.
TRPM3	Transient Receptor Potential Mucolipin 3	a gene that encodes a protein involved in ion transport, particularly calcium ions. It plays a role in sensory processes, including temperature and pain perception.
FAT1	FAT Atypical Cadherin 1	a gene that encodes a protein involved in cell signaling and adhesion. It is associated with the regulation of cell growth and movement and has been linked to various cancers.
CASP8	Caspase 8	a gene that encodes an enzyme involved in the apoptotic cell death pathway. It is a key initiator of apoptosis and plays a role in immune response and cancer.
HRAS	Harvey Rat Sarcoma Viral Oncogene Homolog	a gene that encodes a protein involved in cell signaling and growth regulation. Mutations in HRAS can lead to uncontrolled cell division and are linked to certain cancers.
NOTCH1	Notch Receptor 1	a gene that encodes a receptor involved in cell differentiation, development, and proliferation. It is crucial for processes such as stem cell maintenance and immune cell development.
OS	Overall Survival	a clinical term used to describe the percentage of patients in a clinical trial or study who are still alive after a specific period typically used as a measure of treatment effectiveness in cancer studies.
PFS	Progression-Free Survival	a term used in clinical trials to measure the length of time during and after treatment that a patient lives without the cancer growing or worsening.
CD8	Cluster of Differentiation 8	a glycoprotein found on the surface of cytotoxic T cells. It helps these T cells recognize and destroy infected or cancerous cells.
CD4	Cluster of Differentiation 4	a glycoprotein expressed on the surface of certain immune cells, including T-helper cells (a subset of T cells), macrophages, and dendritic cells. It functions primarily as a co-receptor for the T-cell receptor (TCR) to assist in recognizing antigens presented by MHC class II molecules on antigen-presenting cells.
TIME	Tumor Immune MicroEnvironment	TIME refers to the complex environment around a tumor, including immune cells, blood vessels, and signaling molecules. This microenvironment can influence cancer growth and response to treatment.

Th cells	T-helper cells	a subset of T cells that play a central role in regulating immune responses by assisting other immune cells in recognizing and attacking pathogens or cancer cells.
TNM	Tumor, Node, Metastasis	a system used to stage cancer-based on the size and extent of the primary tumor (T), the involvement of regional lymph nodes (N), and the presence of distant metastasis (M).
COX	Cox Proportional Hazards Model	Cox Regression is a method used in survival analysis to study the effect of multiple variables on the time until an event occurs, where the relationship between predictors and survival time is assumed to be proportional over time. It does not require a specific distribution for survival times and is used to estimate hazard ratios, which represent the relative risk of an event occurring for different values of predictor variables.
TME	Tumor Microenvironment	TME refers to the cellular environment surrounding a tumor, including stromal cells, immune cells, blood vessels, and extracellular matrix. It plays a critical role in cancer progression and therapy response.
FRGs	Ferroptosis-related Genes	FRGs are genes that are associated with ferroptosis, a form of regulated cell death characterized by iron-dependent lipid peroxidation.
ATG5	Autophagy Related 5	a gene that encodes a protein essential for the process of autophagy, a cellular mechanism that degrades and recycles damaged organelles and proteins. It plays a key role in the formation of autophagosomes, which are involved in cellular homeostasis and stress response.
BID	BH3-Interacting Domain Death Agonist	a pro-apoptotic protein that belongs to the Bcl-2 family. It plays a crucial role in regulating apoptosis (programmed cell death) by interacting with other proteins in the mitochondrial pathway.
ACO1	Aconitase 1	an enzyme that catalyzes the conversion of citrate to isocitrate in the citric acid cycle. It also has a role in regulating iron homeostasis and cellular oxidative stress response.
GOT1	Glutamate-Oxaloacetate Transaminase 1	an enzyme involved in amino acid metabolism, specifically the interconversion of glutamate and oxaloacetate. It plays a role in cellular energy production and the metabolism of nitrogen.
GLS2	Glutaminase 2	an enzyme that catalyzes the conversion of glutamine to glutamate, a critical step in cellular metabolism. It plays a role in maintaining cellular energy balance and can be involved in oxidative stress responses.
ALOX15	Arachidonate 15-Lipoxygenase	an enzyme that metabolizes arachidonic acid into bioactive lipids, including hydroperoxyeicosatetraenoic

		acids (HPETEs), which are involved in inflammatory processes and immune responses.
AKR1C3	Aldo-Keto Reductase Family 1 Member C3	an enzyme that catalyzes the reduction of various steroids and other compounds. It is involved in regulating hormone levels, particularly in the metabolism of sex hormones and corticosteroids.
SCO2	Synthesis of Cytochrome c Oxidase 2	a gene involved in the proper assembly of cytochrome c oxidase, a mitochondrial enzyme essential for cellular respiration and energy production. Mutations in SCO2 are associated with mitochondrial disorders.
MAP1LC3A	Microtubule-Associated Protein 1 Light Chain 3 Alpha	a protein that is involved in autophagy. It is conjugated to autophagosomal membranes and helps in the formation of autophagosomes during the process of autophagy.
MAP3K5	Mitogen-Activated Protein Kinase Kinase Kinase 5	a protein kinase that plays a role in signaling pathways related to stress, inflammation, and apoptosis. It is involved in regulating cell survival, differentiation, and response to cellular stress.
NK cells	Natural Killer Cells	a type of immune cell that plays a crucial role in the innate immune system. They are responsible for identifying and killing virus-infected cells, as well as tumor cells, without the need for prior sensitization.
Th2 cell	T-helper 2 Cells	a subset of CD4+ T-helper cells that help activate immune responses by promoting the activity of eosinophils, mast cells, and B cells. They are important in defending against parasitic infections and are involved in allergic responses.
aDCs	Activated Dendritic Cells	dendritic cells that have been stimulated or activated by pathogen signals. They play a central role in initiating immune responses by presenting antigens to T cells and promoting adaptive immunity.
B cells	B Lymphocytes	a type of white blood cell involved in the humoral immune response. They are responsible for producing antibodies, which help neutralize pathogens and mark them for destruction by other immune cells.
iDC	Immature Dendritic Cells	are dendritic cells that have not yet been exposed to an antigen. They are present in peripheral tissues and migrate to lymph nodes to initiate immune responses once they are activated by pathogens.
pDC	Plasmacytoid Dendritic Cells	a specialized type of dendritic cell that plays a key role in antiviral immunity. They produce large amounts of type I interferons in response to viral infections.
IFN	Interferon	are signaling proteins produced by host cells in response to viral infections or other immune triggers. They help regulate the immune response, inhibit viral replication, and modulate the activity of various immune cells.

ARGs	Autophagy-Related Genes	genes involved in the process of autophagy, a cellular mechanism that degrades and recycles damaged organelles, proteins, and other cellular components. They play a key role in maintaining cellular homeostasis and are implicated in diseases such as cancer and neurodegenerative disorders.
USP10	Ubiquitin-Specific Protease 10	an enzyme that removes ubiquitin molecules from proteins, regulating their degradation or function. It plays a role in cellular processes like the cell cycle, DNA repair, and the regulation of immune responses
ATF6	Activating Transcription Factor 6	a transcription factor that plays a key role in the unfolded protein response (UPR), a cellular stress response to misfolded proteins in the endoplasmic reticulum. It helps cells adapt to stress by activating genes that restore cellular homeostasis.
MAPK9	Mitogen-Activated Protein Kinase 9	also known as JNK1, is a member of the MAP kinase family. It is involved in regulating cellular responses to stress, inflammation, apoptosis, and cell differentiation. MAPK9 is particularly activated in response to environmental stresses like UV radiation and oxidative stress.
FOS	Finkel-Biskis-Jinkins Osteosarcoma	a gene that encodes a transcription factor involved in regulating cell growth, differentiation, and survival. It is part of the AP-1 complex and plays a role in signal transduction pathways that respond to stress and mitogenic stimuli.
SPHK1	Sphingosine Kinase 1	an enzyme involved in the metabolism of sphingolipids. It catalyzes the phosphorylation of sphingosine to produce sphingosine-1-phosphate (S1P), which plays a critical role in regulating cell proliferation, migration, and survival.
GRID1	Glutamate Receptor Interacting Protein 1	a gene encoding a protein that interacts with glutamate receptors. It plays a role in the functioning of excitatory synapses in the nervous system and is involved in the regulation of neuronal communication.
IKBKB	Inhibitor of Nuclear Factor Kappa B Kinase Beta	an enzyme involved in the NF-κB signaling pathway, which regulates immune responses, inflammation, and cell survival. It activates NF-κB by phosphorylating IκB proteins, leading to their degradation and allowing NF-κB to translocate to the nucleus and initiate transcription.
RAB24	Ras-Related Protein Rab-24	a member of the Rab family of small GTPases, which regulate intracellular vesicle trafficking. It is involved in processes such as autophagy and cellular stress response.
CFLAR	Caspase-8 and FADD-Like Apoptosis Regulator	a protein that inhibits apoptosis by interacting with the caspase-8 complex. It acts as an anti-apoptotic regulator

		in the extrinsic apoptotic pathway, and its overexpression is often linked to cancer cell survival.
WDR45	WD Repeat Domain 45	a gene that encodes a protein involved in autophagy and neuronal development. Mutations in WDR45 are associated with neurodegenerative diseases, including neurodegeneration with brain iron accumulation (NBIA).
RAF1	Raf Proto-Oncogene Serine/Threonine Kinase 1	a kinase involved in the MAPK/ERK signaling pathway, which regulates cell growth, differentiation, and survival. It is an important component of signaling pathways in cancer.
IRGs	Immunity-Related Genes	genes that are involved in the immune response, particularly in the defense against infections, cancers, and other diseases. They can play a role in inflammation, immune cell activation, and pathogen recognition.
APOD	Apolipoprotein D	a protein that is part of the lipoprotein family. It plays a role in lipid metabolism, and it has been linked to various diseases, including neurodegenerative conditions, cardiovascular diseases, and cancer.
OLR1	Oxidized Low-Density Lipoprotein Receptor 1	a receptor involved in the binding and internalization of oxidized low-density lipoprotein (oxLDL).
STC2	Stanniocalcin 2	a protein involved in regulating calcium and phosphate homeostasis. It is also implicated in cancer progression.
DKK1	Dickkopf WNT Signaling Pathway Inhibitor 1	a protein that inhibits the Wnt signaling pathway, which is crucial for cell proliferation, differentiation, and tissue development.
TNFRSF19	Tumor Necrosis Factor Receptor Superfamily Member 19	a receptor involved in immune cell activation and apoptosis. It plays a role in regulating inflammation and immune responses.
TNFRSF4	Tumor Necrosis Factor Receptor Superfamily Member 4	also known as OX40, is a receptor that regulates T cell activation and survival. It plays a critical role in adaptive immunity and has been studied for its involvement in autoimmune diseases and cancer immunotherapy.
DEFB1	Defensin Beta 1	a gene that encodes beta-defensin, which is an antimicrobial peptide involved in the innate immune response. It has antimicrobial and immunomodulatory properties, playing a role in defense against infections.
CTLA4	Cytotoxic T-Lymphocyte-Associated Protein 4	a protein found on the surface of T cells that acts as an immune checkpoint, negatively regulating T cell activation. It plays a key role in maintaining immune tolerance and preventing autoimmunity.
CTSG	Cathepsin G	an enzyme belonging to the cathepsin family of proteases. It is involved in the breakdown of proteins and plays a role in immune responses, particularly in neutrophil-mediated defense mechanisms.

Tcm cell	Central Memory T cell	a subset of memory T cells that retain the ability to proliferate and differentiate into effector T cells upon re-exposure to an antigen. They play an important role in long-term immunity and immune memory.
Tem cell	Effector Memory T cell	a subset of memory T cells that provide a rapid response to previously encountered pathogens. They circulate in peripheral tissues and are essential for the immediate immune response upon re-infection.
Th1 cell	T-helper 1 cell	a subset of CD4+ T-helper cells that play a key role in promoting cellular immunity. They are involved in the defense against intracellular pathogens, such as viruses and certain bacteria, by activating macrophages and cytotoxic T cells.
Th17 cell	T-helper 17 cell	a subset of CD4+ T-helper cells that secrete interleukin-17 and are involved in inflammatory responses, particularly in defense against extracellular bacteria and fungi. They also play a role in autoimmune diseases.
mRNA	Messenger Ribonucleic Acid	a type of RNA that carries genetic information from the DNA in the nucleus to the ribosome, where it serves as a template for protein synthesis. It plays a central role in gene expression.
CLEC3B	C-Type Lectin Domain Family 3 Member B	a gene that encodes a protein involved in the immune response. It acts as a receptor for pathogens and is involved in the regulation of innate immunity and inflammation.
C6	Complement Component 6	a protein in the complement system, which is part of the innate immune system. It plays a role in immune responses by forming part of the membrane attack complex, which helps destroy pathogens.
CLCN1	Chloride Channel 1	a gene that encodes a chloride channel, which is essential for muscle function. Mutations in CLCN1 are associated with myotonia congenita, a condition characterized by muscle stiffness.
5-MPS	5-Methylthioadenosine Phosphorylase	an enzyme involved in the metabolism of methionine and related compounds. It plays a role in the breakdown of 5'-methylthioadenosine, a byproduct of polyamine biosynthesis.
DGKG	Diacylglycerol Kinase Gamma	an enzyme that catalyzes the conversion of diacylglycerol (DAG) to phosphatidic acid. It plays a role in cellular signaling pathways and the regulation of lipid metabolism.
CA9	Carbonic Anhydrase 9	an enzyme that catalyzes the reversible conversion of carbon dioxide and water to carbonic acid. It is highly expressed in hypoxic tumor cells and is often used as a biomarker in cancer.
EXTL2	Exostosin-Like Glycosyltransferase 2	a gene involved in the synthesis of heparan sulfate, which is important for cell signaling, tissue

		development, and growth. Mutations in EXTL2 are linked to hereditary multiple exostoses.
PGAM1	Phosphoglycerate Mutase 1	an enzyme involved in the glycolysis pathway. It catalyzes the reversible conversion of 3-phosphoglycerate to 2-phosphoglycerate, playing a crucial role in energy metabolism.
TYMS	Thymidylate Synthase	an enzyme involved in the synthesis of thymidine, which is a nucleotide required for DNA replication and repair. TYMS plays a crucial role in cell proliferation and is a target for chemotherapy in cancer treatment.
lncRNA	Long Non-Coding RNA	type of RNA that do not code for proteins but play critical roles in regulating gene expression at various levels, including chromatin modification, transcription, and post-transcriptional processing. They are involved in numerous cellular processes, including development, differentiation, and disease.
DNN-AS1	Dynamin-Related Protein 1-Associated Small RNA 1	a long non-coding RNA (lncRNA) that has been implicated in the regulation of Dynamin-Related Protein 1 (DRP1), which plays a role in mitochondrial fission and cellular energy metabolism.
ALDOC	Aldolase C, Fructose-Bisphosphate	an enzyme that plays a role in glycolysis. It catalyzes the reversible conversion of fructose-1,6-bisphosphate to glyceraldehyde-3-phosphate and dihydroxyacetone phosphate, helping to regulate energy production in tissues like the brain and muscles.
VEGFA	Vascular Endothelial Growth Factor A	a protein that stimulates the formation of blood vessels (angiogenesis) by promoting endothelial cell proliferation and survival. It is essential for wound healing, tissue repair, and cancer development, as tumors require new blood vessels to grow.
HRG	Histidine-Rich Glycoprotein	a protein found in the blood that plays a role in modulating immune responses and regulating the activity of clotting factors. It has functions in the immune system, particularly in interactions with other proteins like complement and antibodies.
PADI3	Peptidyl Arginine Deiminase 3	an enzyme that converts arginine residues in proteins to citrulline through a process known as deimination. This modification can impact protein function and is involved in various biological processes, including skin differentiation.
IGSF11	Immunoglobulin Superfamily Member 11	a protein that is part of the immunoglobulin superfamily and is involved in cell adhesion and signaling. It plays a role in immune responses, especially in the development and functioning of immune cells.
MIPOL1	MIP1-Like Protein 1	a gene encoding a protein involved in various cellular processes, including protein transport and cell

		signaling. It is important for the regulation of cellular functions in the eye and other tissues.
Tfh	T Follicular Helper Cells	a subset of CD4+ T-helper cells that provide support to B cells in germinal centers of lymph nodes, helping them to produce antibodies. They are crucial for the formation of long-lasting immunity and are involved in immune responses to infections and vaccines.
M1 macrophages	Classically Activated Macrophages (M1)	a type of macrophage activated by inflammatory signals such as IFN-γ. They produce pro-inflammatory cytokines and are involved in defending against pathogens and tumors, but excessive activation can lead to chronic inflammation and tissue damage.
M2 macrophages	Alternatively Activated Macrophages (M2)	a type of macrophage involved in tissue repair, wound healing, and the resolution of inflammation. They produce anti-inflammatory cytokines and are important for restoring tissue homeostasis after injury or infection.
M0 macrophages	Naive Macrophages (M0)	resting or naive macrophages that have not yet been exposed to stimuli like pathogens or cytokines. They serve as precursors to M1 and M2 macrophages, which are activated in response to different signals in the immune system.
mRNAsi	mRNA Stemness Index	an index used to quantify the stemness of cancer cells based on the expression levels of certain mRNAs. It is useful in predicting the stem-like properties of cancer cells and their potential to resist treatments, indicating a poor prognosis in cancer.
H2AFZ	H2A Histone Family Member Z	a variant of the histone H2A family. It plays a role in the packaging of DNA into chromatin and is involved in chromatin remodeling and regulation of gene expression.
KPNA2	Karyopherin Subunit Alpha 2	a protein that helps in the nuclear import of proteins by mediating their transport through the nuclear pore complex. It is involved in regulating the import of transcription factors into the nucleus.
NPM3	Nucleophosmin 3	a protein that is involved in various cellular processes such as ribosome biogenesis, cell cycle regulation, and nucleocytoplasmic transport. It is involved in the regulation of transcription and may play a role in cancer.
CCDC92	Coiled-Coil Domain Containing 92	a gene that encodes a protein with a coiled-coil domain, which is involved in protein-protein interactions. The function of CCDC92 is not completely understood, but it may have roles in cellular signaling or structural integrity.
GAS1	Growth Arrest-Specific 1	a protein involved in growth arrest and may play a role in cell cycle regulation. It is thought to act as a tumor

		suppressor gene, inhibiting the growth of certain cancer cells.
IGLV2	Immunoglobulin Lambda Variable 2	a gene that encodes a variable region of lambda light chains of antibodies. It plays a role in immune responses by contributing to the antigen-binding specificity of immunoglobulins.
TWIST2	Twist Family BHLH Transcription Factor 2	a transcription factor involved in embryonic development and the formation of mesodermal tissues. It is important for regulating cell differentiation and has been implicated in tumor metastasis.
CLEC3B	C-Type Lectin Domain Family 3 Member B	a protein involved in the immune system's recognition of pathogens. It plays a role in immune responses and has been linked to the regulation of inflammation.
CCL22	C-C Motif Chemokine Ligand 22	a chemokine that acts as a signal to attract specific immune cells, particularly T-regulatory cells (Tregs). It plays a role in immune tolerance and inflammation.
TPSAB1	Tryptase Alpha/Beta 1	an enzyme secreted by mast cells involved in immune responses. It is associated with allergic reactions and has been linked to various inflammatory conditions.
TSPN11	Thrombospondin 1	a glycoprotein that plays a role in cell adhesion, angiogenesis, and wound healing. It is involved in regulating vascular endothelial growth and has implications in cancer progression.
Tgd	T Gamma Delta (Tgd) Cells	a subset of T lymphocytes that express gamma and delta chains instead of the usual alpha and beta chains. They play an important role in innate immune responses, particularly in fighting infections and tumors.
m6A-RLPS	N6-Methyladenosine RNA Reader-Like Proteins	proteins that read and interpret the m6A modification on RNA molecules. This modification is a key post-transcriptional modification in RNA, affecting RNA stability, splicing, and translation.
LINC01644	Long Intergenic Non-Coding RNA 01644	a long non-coding RNA (lncRNA) involved in the regulation of gene expression and cellular processes. lncRNAs like LINC01644 have been implicated in cancer progression and other diseases.
LINC01410	Long Intergenic Non-Coding RNA 01410	a long non-coding RNA involved in regulating gene expression. It is associated with various processes, including tumor progression and immune response regulation.
LINC00630	Long Intergenic Non-Coding RNA 00630	a long non-coding RNA that has been implicated in the regulation of gene expression, particularly in cancer progression and other diseases.
LINC00992	Long Intergenic Non-Coding RNA 00992	a long non-coding RNA that regulates various cellular processes. It has been associated with the development of certain tumors and is involved in gene expression regulation.

JPX	Jpx RNA	a long non-coding RNA that is involved in the regulation of X-chromosome inactivation. It plays a role in the process where one of the two X chromosomes in female mammals is randomly silenced.
LINC01775	Long Intergenic Non-Coding RNA 01775	a long non-coding RNA that is involved in the regulation of gene expression and is being studied for its roles in cancer and other diseases.
CAFs	Cancer-Associated Fibroblasts	a type of fibroblast that are found in the tumor microenvironment. They are involved in tumor progression, angiogenesis, and the remodeling of the extracellular matrix. CAFs play an important role in cancer metastasis and chemoresistance.
AFOC-DEGs	arecoline-associated fibrosis-related genes-differential gene expression	Genes with altered expression in response to arecoline, a compound linked to fibrosis, particularly in conditions like **oral submucous fibrosis**. These genes are involved in the fibrotic process and help understand its molecular mechanisms.
AFOC	arecoline-associated fibrosis-related genes	Genes that are involved in the development of fibrosis triggered by arecoline; a compound found in betel nuts. These genes play a role in the fibrotic process, which can lead to conditions such as oral submucous fibrosis.
PLAU	Plasminogen Activator, Urokinase	an enzyme that plays a key role in the breakdown of fibrin in blood clots (fibrinolysis). It activates plasminogen into plasmin, which dissolves fibrin clots. It is involved in wound healing, tissue remodeling, and cancer metastasis.
IL1A	Interleukin 1 Alpha	a pro-inflammatory cytokine that is involved in the regulation of immune and inflammatory responses. It plays a central role in fever, immune activation, and the pathogenesis of inflammatory diseases.
SPP1	Secreted Phosphoprotein 1	also known as osteopontin, is a protein involved in cell signaling, immune responses, and tissue remodeling. It plays a role in inflammation, cancer metastasis, and wound healing.
CCl11	C-C Motif Chemokine Ligand 11	a chemokine that attracts eosinophils to sites of inflammation. It is involved in allergic responses and has roles in immune system regulation, particularly in asthma and chronic inflammatory diseases.
TER	Telomerase	a component of telomerase, an enzyme that extends the telomeres at the ends of chromosomes, maintaining chromosome integrity. It plays a key role in cellular aging and is often dysregulated in cancer cells.
COL1A2	Collagen Type I Alpha 2 Chain	a gene that encodes the alpha-2 chain of type I collagen, a major component of the extracellular matrix. It is involved in providing structural support to tissues and is important for wound healing and tissue repair.

FAM122C	Family With Sequence Similarity 122 Member C	a protein that may be involved in various cellular processes, although its specific function is still under investigation. It is thought to play a role in cell signaling and possibly in cancer development.
RNF157	Ring Finger Protein 157	a ubiquitin ligase that may regulate the protein degradation pathway by tagging proteins for degradation. It plays a role in regulating cellular homeostasis and may be involved in cancer progression.
RANBP17	RAN Binding Protein 17	involved in nuclear-cytoplasmic transport and may play a role in the regulation of cellular signaling. It is a binding partner of Ran, a small GTPase, and is involved in nuclear transport.
SOWAHA	Sodium-Dependent Organic Anion Transporter	refers to a group of transporters that help in the transport of organic anions across cellular membranes, relying on the sodium gradient. They are involved in various physiological processes, including drug metabolism and excretion.
KIAA1211	KIAA1211 Gene	a gene that encodes a protein of unknown function. It has been implicated in certain cancer-related pathways, but its exact role in cellular functions remains to be fully elucidated.
RIPPLY2	Ripply Transcriptional Repressor 2	is involved in the regulation of gene expression during embryonic development, particularly in mesodermal and neural differentiation. It plays a role in cell fate decisions and development.
INSL3	Insulin-Like 3	a peptide hormone that is primarily involved in the development of male reproductive tissues. It plays a role in testicular descent and is also involved in the regulation of vascular tone.
DNAH1	Dynein Axonemal Heavy Chain 1	a protein involved in the structure and function of ciliary dynein, a motor protein that helps in the movement of cilia. Mutations in DNAH1 are associated with primary ciliary dyskinesia, a disorder affecting cilia function.
DEGs	Differentially Expressed Genes	genes whose expression levels are significantly altered under specific conditions, such as disease, treatment, or experimental manipulation. Analyzing DEGs helps to understand molecular mechanisms in diseases like cancer.
CTSG	Cathepsin G	an enzyme released by neutrophils during immune responses. It has protease activity and plays a role in digestion of foreign particles, tissue remodeling, and inflammation.
TNFRSF4	Tumor Necrosis Factor Receptor Superfamily Member 4	also known as OX40, is a receptor involved in T-cell activation. It plays a crucial role in regulating immune responses and inflammatory diseases.

IGLVQ-44	Immunoglobulin Lambda Variable Region Q-44	a gene that encodes a variable region of lambda light chains in antibodies. It is involved in the immune system's ability to recognize and respond to pathogens.
STC2	Stanniocalcin 2	a protein involved in calcium and phosphate homeostasis. It plays a role in bone metabolism and has been implicated in the regulation of tumor growth.
CCL22	C-C Motif Chemokine Ligand 22	a chemokine involved in the attraction of regulatory T cells to sites of inflammation and has a role in immune tolerance and immune modulation.
CIBERSORT	Cell Type Identification by Estimating Relative Subsets Of RNA Transcripts	an analytical tool used to estimate the abundance of specific cell types in a tissue sample based on gene expression data. It is particularly used in tumor microenvironment analysis to understand immune infiltration.
SGs	Shelterin Complex Genes	genes that encode proteins forming the shelterin complex, which protects the ends of chromosomes (telomeres) from degradation and improper repair. These genes play a critical role in maintaining telomere stability, regulating telomere length, and preventing chromosome fusion
TRF1	Telomeric Repeat-binding Factor 1	TRF1 is a protein that binds to the telomeric regions of chromosomes and plays a role in telomere maintenance, preventing telomere elongation and regulating chromosome stability.
TRF2	Telomeric Repeat-binding Factor 2	TRF2 is another protein involved in telomere protection and stability. It prevents the chromosome ends from being recognized as DNA damage, thus protecting telomeres from degradation.
RAP1	Repressor Activator Protein 1	RAP1 is involved in telomere maintenance and DNA repair, regulating telomeric DNA structure and length to ensure proper chromosome protection during cell division.
TPP1	Telomere Protection 1	TPP1 is a protein that works with other telomere-binding factors to protect and maintain telomeres, ensuring chromosomal stability and preventing telomere attrition.
POT1	Protection of Telomeres 1	POT1 is a protein that binds to the single-stranded DNA at telomeres, providing protection against DNA damage and regulating telomere length.
TIN2	TRF1-interacting Nuclear Factor 2	a protein that interacts with TRF1 and TRF2, helping maintain telomere structure and chromosomal stability during cell division.
APCs	Antigen-Presenting Cells	Immune cells (such as dendritic cells, macrophages, and B cells) that capture, process, and present antigens on their surface using major histocompatibility complex (MHC) molecules, activating T cells and triggering an immune response.

HLA	Human Leukocyte Antigen	HLAs are proteins on the surface of cells that are involved in the immune response by presenting foreign antigens to T cells. They are crucial for immune system function.
pDC	Plasmacytoid Dendritic Cells	specialized immune cells that produce high amounts of type I interferons in response to viral infections and are involved in regulating immune responses.
TIL	Tumor-Infiltrating Lymphocytes	TILs are immune cells, mainly T cells, that infiltrate tumors and play a role in anti-tumor immunity. Their presence in tumors is often associated with better prognosis in cancers.
IPS score	Immune Profile Score	IPS score is a measure used to evaluate the immune cell profile in the tumor microenvironment. It is used to assess the potential for immune responses in cancer immunotherapy.
IC50	Half Maximal Inhibitory Concentration	IC50 is a measure used to determine the effectiveness of a substance in inhibiting a biological or biochemical function, such as drug efficacy in cancer or enzyme inhibition.
SHMT2	Serine Hydroxymethyltransferase 2	an enzyme involved in the folate metabolism pathway and plays a role in one-carbon metabolism, essential for DNA synthesis and cell division.
HPRT1	Hypoxanthine-guanine Phosphoribosyltransferase 1	an enzyme that is involved in the purine salvage pathway, critical for maintaining nucleotide balance in cells. Mutations in HPRT1 lead to Lesch-Nyhan syndrome.
POLD2	Polymerase Delta 2	a subunit of DNA polymerase delta, involved in DNA replication and repair processes, particularly during DNA synthesis and strand displacement.
HADHB	Hydroxyacyl-CoA Dehydrogenase Beta Subunit	involved in fatty acid oxidation, particularly in the mitochondria, and is essential for energy production and metabolic processes.
POLE3	Polymerase epsilon 3	POLE3 is a subunit of DNA polymerase epsilon, which is involved in DNA replication and repair processes, contributing to the maintenance of genomic stability.
ADK	Adenosine Kinase	ADK is an enzyme that plays a role in regulating adenosine levels within cells, which is important for energy metabolism, signal transduction, and neurological function.
ATIC	5'-Phosphoribosyl-5'-Adenosine Cyclase	ATIC is involved in the purine biosynthesis pathway and contributes to nucleotide metabolism necessary for cell growth and DNA synthesis.
ADA	Adenosine Deaminase	ADA is an enzyme involved in the purine metabolism pathway, converting adenosine to inosine. Deficiency in ADA leads to severe combined immunodeficiency (SCID).

GNPDA1	Glucosamine-6-Phosphate Deaminase 1	GNPDA1 is involved in glucosamine metabolism, which plays a role in glycosylation and sugar metabolism in the body.
EMT	Epithelial-Mesenchymal Transition	EMT is a process in which epithelial cells lose their polarity and adhesion properties, gaining mesenchymal characteristics, which is crucial in development, wound healing, and cancer metastasis.
AREG	Amphiregulin	AREG is a growth factor that binds to EGFR and is involved in cell growth, repair, and tissue regeneration. It is important in various cancers and inflammatory diseases.
DKK1	Dickkopf-1	DKK1 is a protein that inhibits the Wnt signaling pathway, which is involved in cellular differentiation and cancer progression. It plays a role in bone metabolism and neural development.
PLOD2	Procollagen-Lysine, 2-Oxoglutarate 5-Dioxygenase 2	PLOD2 is an enzyme that catalyzes the hydroxylation of lysine residues in collagen, contributing to collagen stability and extracellular matrix formation.
SFRP1	Secreted Frizzled-Related Protein 1	SFRP1 is a Wnt signaling pathway inhibitor and plays a role in regulating cellular proliferation and differentiation. It is involved in cancer suppression and development.
TNFRSF11B	Tumor Necrosis Factor Receptor Superfamily Member 11B	TNFRSF11B, also known as osteoprotegerin (OPG), is involved in the regulation of bone metabolism and the immune response, particularly in osteoclastogenesis.
GPX7	Glutathione Peroxidase 7	GPX7 is an enzyme that protects cells from oxidative stress by reducing reactive oxygen species (ROS) and plays a role in cell survival and cancer prevention.
COL5A3	Collagen Type V Alpha 3 Chain	COL5A3 is a gene encoding a component of type V collagen, which is involved in extracellular matrix formation and plays a role in tissue structural integrity.
Tgd cells	T Gamma Delta Cells	Tgd cells are a subset of T cells that express gamma delta T-cell receptors. They are involved in immune surveillance, particularly against pathogens and tumors.
PGAM5	Phosphoglycerate Mutase Family Member 5	A mitochondrial protein involved in regulating mitochondrial dynamics and cell survival under stress conditions.
SMN1	Survival of Motor Neuron 1	A gene that encodes a protein essential for the survival of motor neurons.
FADD	Fas-associated Death Domain	A protein involved in apoptotic signaling that interacts with Fas and other receptors to initiate programmed cell death.
KIAA1191	KIAA1191 (also known as C9orf92)	A gene with an unknown function, but its expression has been linked to cellular stress response and cancer progression.

CD56 bright cells	Bright CD56+ Natural Killer Cells	A subset of natural killer (NK) cells characterized by high expression of CD56 and typically associated with cytokine production rather than cytotoxicity.
CD56 dim cells	Dim CD56+ Natural Killer Cells	A subset of natural killer (NK) cells characterized by lower expression of CD56 and primarily involved in cytotoxicity against infected or tumor cells.
ERS	Endoplasmic reticulum stress	A condition where the endoplasmic reticulum (ER) experiences stress due to the accumulation of unfolded or misfolded proteins, triggering adaptive responses and potentially leading to cell death.
IBSP	Integrin-binding Sialoprotein	A protein involved in bone mineralization and osteoblast adhesion. It plays a role in the extracellular matrix in bone tissue.
RDM1	Recombination Activating Gene 1 (RAG1)	A gene involved in DNA repair and V(D)J recombination, which is essential for the development of immune diversity in T and B cells.
RBP4	Retinol-binding Protein 4	A protein that binds and transports vitamin A (retinol) in the bloodstream, playing a role in vision and metabolic health.
CRLs	Cuproptosis-related IncRNAs	These are long non-coding RNAs (lncRNAs) that are associated with cuproptosis, a newly discovered form of cell death induced by copper toxicity. These lncRNAs play a role in regulating copper metabolism and modulating the cell's response to copper overload, which can lead to cell death.
FAM27E3	Family with Sequence Similarity 27 Member E3	A gene with limited functional characterization, involved in cell signaling and development.
MYOSLID	Myosin, Smooth Muscle, Light Chain Kinase, Downstream	A protein related to smooth muscle function and cytoskeletal organization.
LINC02367	Long Intergenic Non-Coding RNA 02367	A lncRNA that is involved in the regulation of gene expression and cellular processes such as proliferation and differentiation.
6-GPS	6-gene cancer stem cell-related prognostic signature	a set of six genes associated with cancer stem cells used to predict prognosis and survival outcomes in cancer patients. It helps in identifying aggressive cancers and guiding personalized treatments based on tumor behavior and resistance to therapies.
ADM	Adrenomedullin	A peptide involved in the regulation of vascular tone, blood pressure, and fluid balance. It also plays a role in inflammation and immune response.
RPL35A	Ribosomal Protein L35a	A component of the ribosome involved in protein synthesis and cellular growth.
PGK1	Phosphoglycerate Kinase 1	An enzyme involved in glycolysis, catalyzing the conversion of 1,3-bisphosphoglycerate to 3-phosphoglycerate, and playing a role in energy production.

POLR1D	RNA Polymerase I Subunit D	A subunit of RNA polymerase I, which is responsible for ribosomal RNA synthesis in the nucleolus.
P4HA1	Prolyl 4-Hydroxylase Alpha Subunit 1	An enzyme involved in the post-translational modification of collagen, particularly in proline hydroxylation, which is essential for collagen stability.
PTGR1	Prostaglandin-Two-Dioxygenase-Related 1	An enzyme involved in the metabolism of prostaglandins, which are involved in inflammatory responses.
CD133	Cluster of Differentiation 133	A cell surface marker used to identify stem cells in various tissues, particularly in cancer stem cells.
JAK1	Janus Kinase 1	A kinase involved in cytokine signaling and immune response regulation. Mutations in JAK1 can lead to immune disorders and cancer.
HIF1α	Hypoxia-Inducible Factor 1 Alpha	A transcription factor that regulates the cellular response to hypoxia (low oxygen levels), playing a crucial role in angiogenesis and tumor growth.
CCND1	Cyclin D1	A protein involved in cell cycle regulation that promotes the transition from G1 to S phase, critical for cell proliferation. Its dysregulation is linked to cancer.
TMB score	Tumor Mutational Burden score	A measure of the number of mutations found in a tumor's genome. A higher TMB score often correlates with a greater likelihood of response to immunotherapy.
AFAP-AS1	Actin Filament Associated Protein 1-Associated Small RNA 1	A long non-coding RNA (lncRNA) associated with actin filament dynamics has been implicated in cancer progression and metastasis.
ALMS1-IT1	Alstrom Syndrome 1-Intergenic Transcript 1	A lncRNA related to Alstrom syndrome is involved in regulating various cellular functions such as cell growth and differentiation.
HLA-F-AS1	HLA-F Antisense RNA 1	A lncRNA is involved in the regulation of immune responses, particularly in the context of HLA-F gene expression, which plays a role in immune surveillance.
LINC-PINT	Long Intergenic Non-Coding RNA-PINT	A lncRNA that acts as a tumor suppressor by regulating gene expression and has been linked to various cancers.
LINC00958	Long Intergenic Non-Coding RNA 00958	A lncRNA is associated with cancer progression, specifically regulating genes related to cell proliferation and metastasis.
NPSR1-AS1	Neuropeptide S Receptor 1 Antisense RNA 1	A lncRNA modulates the expression of NPSR1, which is involved in immune regulation and could affect neuroimmune interactions in disease.
PRKG1-AS1	Protein Kinase cGMP-Dependent 1 Antisense RNA 1	A lncRNA is involved in the regulation of PRKG1, which plays a role in vascular smooth muscle relaxation and cellular signaling.
WDFT3-AS2	WD Repeat Domain, False-Tandem Repeat 3 Antisense RNA 2	A lncRNA is involved in regulating the WDFT3 gene, which is important for various cellular functions, including differentiation and growth.

KANSL1-AS1	KAT8 Regulated Lysine Acetyltransferase 1 Antisense RNA 1	A lncRNA regulates the KANSL1 gene, which is involved in chromatin remodeling and has been linked to developmental disorders and cancer.
LINC00567	Long Intergenic Non-Coding RNA 00567	A lncRNA involved in cancer progression, particularly in breast cancer, by regulating cell proliferation and migration.
LINC00689	Long Intergenic Non-Coding RNA 00689	A lncRNA associated with regulating cancer stem cells and tumor microenvironment in various cancers.
LINC00877	Long Intergenic Non-Coding RNA 00877	lncRNA involved in the regulation of cellular growth and metastasis, with potential links to cancer progression.
LINC01191	Long Intergenic Non-Coding RNA 01191	A lncRNA implicated in cancer metastasis and cell migration processes.
MDSC	Myeloid-Derived Suppressor Cells	A population of immature myeloid cells that suppress immune responses, promoting tumor growth and metastasis by inhibiting T cell activity.
ZFAS1	Zinc Finger Antisense 1	A long non-coding RNA (lncRNA) involved in the regulation of gene expression and tumor progression, often associated with cancer.
TNFRSF10A-AS1	TNF Receptor Superfamily Member 10A Antisense RNA 1	A lncRNA that regulates the expression of the TNFRSF10A gene, involved in apoptosis and cell survival pathways.
LINC00847	Long Intergenic Non-Coding RNA 00847	A lncRNA implicated in gene regulation related to tumor growth and cancer metastasis.
IER3-AS1	Immediate Early Response 3 Antisense RNA 1	A lncRNA that is associated with cellular stress response, involved in cell survival and apoptosis.
ZJPX	Zinc Finger Protein X	A lncRNA involved in the regulation of gene expression and potentially playing a role in cancer biology.
LRGs	Lysosome-Related Genes	Genes associated with the lysosomal function, including cellular degradation processes and related to diseases like lysosomal storage disorders.
SLC46A3	Solute Carrier Family 46 Member 3	A transporter protein involved in the transport of nutrients and metabolites across the cell membrane.
MANBA	Mannosidase Beta A	An enzyme involved in glycosylation and carbohydrate processing, with relevance to diseases like lysosomal storage disorders.
NEU1	Neuraminidase 1	An enzyme that removes sialic acid residues from glycoproteins, involved in cellular signaling and immune regulation.
SDCBP	Syntrophin-Dystrobrevin Binding Protein	A protein involved in muscle function and cell signaling, particularly in the neuromuscular junction.
BRI3	BRI3 Gene	A gene associated with neuronal function and cellular stress responses.
TMEM175	Transmembrane Protein 175	A transmembrane protein involved in maintaining ion balance and associated with diseases like Parkinson's disease.

GPC1	Glypican 1	A cell membrane-associated heparan sulfate proteoglycan involved in cell growth and cancer progression.
SFTPB	Surfactant Protein B	A protein important for the lung surfactant system, aiding in lung function and pulmonary health.
TPP1	Tripeptidyl-Peptidase 1	An enzyme important for protein degradation in the lysosome, associated with neuronal function and cognitive disorders.
BGN	Biglycan	A proteoglycan involved in the extracellular matrix and cellular signaling, implicated in various cancer and inflammatory diseases.
TMEM192	Transmembrane Protein 192	A membrane protein involved in cell signaling and protein transport across membranes.
ssGSEA	Single sample gene set enrichment analysis	A computational method used to evaluate the activity of predefined gene sets in individual samples, helping to assess biological processes in clinical settings.
CAFs	Cancer-Associated Fibroblasts	Fibroblasts within the tumor microenvironment that support tumor growth, invasion, and therapy resistance.
ACTN2	Alpha-Actinin-2	A protein involved in actin filament crosslinking and muscle contraction, playing a role in muscle function.
AQP1	Aquaporin 1	A water channel protein involved in water transport across cell membranes, especially in kidneys and red blood cells.
IL10	Interleukin 10	A cytokine with anti-inflammatory properties, important for regulating immune responses and autoimmune diseases.
SLC2A3	Solute Carrier Family 2 Member 3	A glucose transporter responsible for glucose uptake into vascular endothelial cells.
TIMP4	Tissue Inhibitor of Metalloproteinases 4	A protein that inhibits the activity of matrix metalloproteinases (MMPs), involved in the regulation of extracellular matrix turnover.
HLA-A	Human Leukocyte Antigen A	A class I MHC protein involved in presenting peptides to the immune system, important for immune recognition.
HLA-B	Human Leukocyte Antigen B	A class I MHC protein that plays a key role in immune response by presenting antigens to cytotoxic T cells.
HLA-C	Human Leukocyte Antigen C	A class I MHC protein that participates in presenting antigens to the immune system and plays a role in immune regulation.
HLA-DMA	Human Leukocyte Antigen DMA	A class II MHC protein involved in presenting exogenous antigens to the immune system, especially to helper T cells.
RPL23AP7	Ribosomal Protein L23A Pseudogene 7	A pseudogene related to ribosomal protein L23A, potentially involved in cellular stress responses and gene regulation.

USP34	Ubiquitin-Specific Protease 34	An enzyme that removes ubiquitin from proteins, playing a role in protein degradation and cell cycle regulation.
ASXL3	Additional Sex Combs-Like 3	A protein involved in chromatin remodeling, and mutations in ASXL3 are linked to neurodevelopmental disorders.
LRRTM1	Leucine-Rich Repeat Transmembrane Neuronal 1	A protein involved in synaptic signaling and neuronal development, often associated with neurodevelopmental diseases.
TPTE	Tetraspanin-Containing Protein	A membrane protein involved in various cellular processes, including signal transduction and cell adhesion.
PIK3CA	Phosphoinositide-3-Kinase Catalytic Subunit Alpha	An enzyme involved in the PI3K/AKT signaling pathway, important in cell growth, proliferation, and survival; frequently mutated in cancer.
ATRX	Alpha-Thalassemia/Mental Retardation Syndrome X-Linked	A gene involved in chromatin remodeling, mutations are linked to mental retardation and cancer.
CACNA1C	Calcium Voltage-Gated Channel Subunit Alpha1 C	A gene encoding a subunit of the calcium channel, critical for cardiac and neuronal function.
KMT2E	Lysine Methyltransferase 2E	A histone methyltransferase involved in the regulation of gene expression and cell differentiation.
AJUBA	Ajuba LIM Protein	A protein involved in cellular signaling, adhesion, and migration.
CDKN2A	Cyclin-Dependent Kinase Inhibitor 2A	A tumor suppressor gene that encodes p16INK4a, involved in cell cycle regulation and frequently mutated in cancer.
NEB	Nebulin	A protein involved in muscle contraction and the structural integrity of sarcomeres in muscle cells.
PD-1	Programmed Cell Death Protein 1	A receptor on T cells that regulates immune response and is a target for immunotherapy in cancer.
PR-DE-FRGs	prognostic-related differentially expressed ferroptosis-related genes	A set of genes related to fibrosis that can help predict prognosis in diseases such as liver or lung fibrosis.
CISD2	CDGSH Iron-Sulfur Domain 2	A protein involved in mitochondrial function and cellular homeostasis, associated with neurodegenerative diseases.
DDIT4	DNA Damage Inducible Transcript 4	A stress-responsive protein involved in regulating cell survival and apoptosis in response to DNA damage.
CA9	Carbonic Anhydrase 9	An enzyme expressed in hypoxic tumors, is involved in acid-base regulation and tumor progression.
ALOX15	Arachidonate 15-Lipoxygenase	An enzyme involved in lipid metabolism, playing a role in inflammation and immune responses.
ATG5	Autophagy-Related 5	A protein is involved in autophagy, a process crucial for cellular homeostasis and the degradation of damaged proteins.

BECN1	Beclin 1	A protein is involved in the initiation of autophagy and regulating the lysosomal degradation of cellular components.
BNIP3	BCL2/adenovirus E1B 19kDa Interacting Protein 3	A protein that plays a role in mitochondrial autophagy, apoptosis, and is associated with cellular stress responses.
PRDX5	Peroxiredoxin 5	An enzyme that reduces reactive oxygen species (ROS) and is involved in cell protection from oxidative stress.
MAP1LC3A	Microtubule-Associated Protein 1 Light Chain 3 Alpha	A protein involved in autophagy, playing a key role in vesicle formation and degradation of cellular components.
KM analysis	Kaplan-Meier Analysis	A statistical method used to estimate survival functions and time-to-event data, commonly used in clinical research.
FLT3	Fms-Like Tyrosine Kinase 3	A receptor tyrosine kinase is involved in hematopoiesis and stem cell differentiation, with mutations linked to leukemia.
IGLV4.60	Immunoglobulin Lambda Variable 4-60	A gene that encodes a variable region of the lambda light chain in antibodies.
LINC00861	Long Intergenic Non-Coding RNA 00861	A lncRNA is involved in gene regulation and is associated with cancer progression.
MS4A2	Membrane-Spanning 4-Domains Subfamily A Member 2	A cell surface protein is involved in immune cell signaling, particularly in mast cells and B cells.
GALR2	Galanin Receptor 2	A receptor involved in neurological and endocrine regulation, affecting mood and appetite.
LINC01508	Long Intergenic Non-Coding RNA 01508	A lncRNA is involved in the regulation of gene expression related to tumor progression.
IGKV1D.8	Immunoglobulin Kappa Variable 1D-8	A variable region gene in immunoglobulin kappa light chains, is involved in immune response.
IGLV1.36	Immunoglobulin Lambda Variable 1-36	a gene that encodes the variable region of the lambda light chain in immunoglobulins (antibodies). This variable region is crucial for antibody diversity and the immune response, as it plays a role in antigen binding during immune reactions.
PARDEGs	Polyamine-related differentially expressed genes	These are genes whose expression is significantly altered in response to changes in polyamine metabolism. Polyamines are small molecules that play crucial roles in cell growth, proliferation, and differentiation, and their dysregulation is associated with various diseases, including cancer. PARDEGs may be involved in these processes, making them potential biomarkers or therapeutic targets.
CKS2	Cyclin-Dependent Kinase Subunit 2	A protein involved in regulating the cell cycle, particularly in the activation of cyclin-dependent kinases (CDKs).
RIMS3	Regulating Synaptic Membrane Exocytosis 3	A protein that is involved in synaptic vesicle exocytosis, helping in neurotransmitter release.

TRAC	T Cell Receptor Alpha Constant	A component of the T cell receptor (TCR), crucial for T cell activation and immune response.
FMOD	Fibromodulin	A protein involved in extracellular matrix formation and the regulation of collagen fibril organization.
CALML5	Calmodulin-Like Protein 5	A calcium-binding protein involved in cell signaling and regulation of cellular functions like proliferation.
SPINK7	Serine Peptidase Inhibitor, Kazal Type 7	A serine protease inhibitor that regulates proteolytic activity in the pancreas and other tissues.
STC2	Stanniocalcin 2	A protein that regulates calcium and phosphate metabolism, playing a role in bone health and tumorigenesis.
TBC1D2	TBC1 Domain Family Member 2	A protein involved in regulating vesicle trafficking and cellular signaling.
ADM	Adrenomedullin	A peptide hormone involved in vascular tone regulation and the immune response.
NDRG1	N-Myc Downstream Regulated Gene 1	A protein associated with cell differentiation, growth, and stress response, often involved in cancer progression.
OLR1	Oxidized Low-Density Lipoprotein Receptor 1	A receptor involved in lipid metabolism and the regulation of inflammatory responses.
PDGFA	Platelet-Derived Growth Factor Alpha	A growth factor involved in cell proliferation, angiogenesis, and tissue repair.
ANO1	Anoctamin 1	A calcium-activated chloride channel involved in muscle contraction and ion transport.
PDCD1	Programmed Cell Death 1	A receptor on T cells that plays a critical role in immune checkpoint regulation, inhibiting T cell activation to maintain immune homeostasis.
CD96	Cluster of Differentiation 96	A cell surface protein involved in immune cell interactions and inhibiting immune responses.
TIGIT	T cell Immunoreceptor with Ig and ITIM Domains	An immune checkpoint receptor that inhibits T cell activation and promotes immune tolerance.
LAG3	Lymphocyte-Activation Gene 3	A protein that inhibits T cell activation and is involved in immune regulation.
PVR	Poliovirus Receptor	A cell surface receptor involved in immune cell signaling, particularly in T cell activation and cancer immunotherapy.
SRGs	Senescence-related genes	A set of genes involved in cellular senescence, a state of permanent growth arrest that can contribute to aging and cancer progression.
CDK1	Cyclin-Dependent Kinase 1	A protein kinase involved in the regulation of the cell cycle, particularly in the transition from G2 to M phase.
G6PD	Glucose-6-Phosphate Dehydrogenase	An enzyme involved in the pentose phosphate pathway, critical for oxidative stress protection and red blood cell function.
IL1A	Interleukin 1 Alpha	A pro-inflammatory cytokine involved in the regulation of the immune response and inflammation.

MAD2L1	Mitotic Arrest Deficient 2 Like 1	A protein that regulates the mitotic checkpoint, ensuring accurate chromosome segregation during cell division.
PDCD10	Programmed Cell Death 10	A protein involved in cell survival and apoptosis, playing a role in vascular development and brain function.
PTTG1	Pituitary Tumor-Transforming Gene 1	A gene involved in cell cycle regulation, particularly in the control of chromosome separation during mitosis.
VEGFA	Vascular Endothelial Growth Factor A	A growth factor that promotes angiogenesis (formation of new blood vessels), crucial for tumor growth and wound healing.
p21	Cyclin-Dependent Kinase Inhibitor 1	A cell cycle inhibitor that regulates cellular response to stress and DNA damage, playing a role in tumor suppression.
SA-β-Gal	Senescence-Associated Beta-Galactosidase	A biomarker used to detect cellular senescence, an important process in aging and the regulation of cell proliferation.
CGNL1	Cingulin-Like 1	A protein involved in the regulation of tight junctions in cells, particularly important for maintaining cellular integrity and epithelial barrier function.
VWCE	Viral Wound-Response Cysteine-Rich Protein	A protein associated with the wound healing process and possibly involved in immune responses.
ZFP42	Zinc Finger Protein 42	A transcription factor that plays a role in embryonic stem cell regulation and is involved in self-renewal and differentiation.
PCA differences	Principal Component Analysis Differences	A statistical method used to reduce the dimensionality of data while preserving most of its variance. PCA is commonly used to analyze gene expression data and identify key differences between experimental conditions.
ESTIMATE	Estimation of STromal and Immune cells in MAlignant Tumor tissues using Expression data	A computational method to estimate the levels of stromal and immune cell infiltration in tumor tissues based on gene expression profiles.
THAP9-AS1	THAP9 Antisense RNA 1	A long non-coding RNA (lncRNA) that is involved in gene regulation and may play a role in tumorigenesis and other biological processes.
STARD4-AS1	STARD4 Antisense RNA 1	A lncRNA involved in regulating the expression of the STARD4 gene, which is involved in lipid metabolism and other cellular functions.
WDFY3-AS2	WDFY3 Antisense RNA 2	A long non-coding RNA that may play a role in cellular processes and is associated with immune regulation.
CDKN2A-DT	CDKN2A Divergent Transcript	A long non-coding RNA associated with the CDKN2A gene, which is involved in cell cycle regulation and tumor suppression.

GCC2-AS1	GCC2 Antisense RNA 1	A lncRNA that regulates the expression of the GCC2 gene, which is involved in vesicle trafficking and cellular processes.
CD5	Cluster of Differentiation 5	A cell surface glycoprotein found on T cells and B cells, involved in immune response and T cell activation.
IL12RB2	Interleukin 12 Receptor Subunit Beta 2	A receptor subunit for interleukin 12 (IL-12), involved in the activation of T cells and the regulation of immune responses.
TIDE	Tumor Immune Dysfunction and Exclusion	A computational framework used to predict how tumors might escape the immune system, evaluating the effectiveness of immune checkpoint inhibitors in cancer therapy.
RPN1	Ribosomal Protein N1	A component of the ribosome, involved in protein synthesis.
OXSM	Oxidosqualene Cyclase, Mitochondrial	An enzyme involved in the biosynthesis of sterols and other lipids in mitochondria.
NDUFA11	NADH:Ubiquinone Oxidoreductase Subunit A11	A subunit of complex I in the mitochondrial electron transport chain, involved in energy production.
GYS1	Glycogen Synthase 1	An enzyme responsible for the synthesis of glycogen from glucose, essential for energy storage.
SLC3A2	Solute Carrier Family 3 Member 2	A transporter protein involved in the transport of amino acids and small peptides.
SreBP	Sterol Regulatory Element-Binding Protein	A transcription factor that regulates lipid metabolism, including cholesterol and fatty acid synthesis, in response to lipid levels.
GPX4	Glutathione Peroxidase 4	An enzyme that protects cells from oxidative damage by reducing lipid peroxides and preventing ferroptosis.
FcγR	Fc Gamma Receptor	A receptor for the Fc region of immunoglobulin G (IgG), important in immune response.
HADb	Human Autophagy Database	A resource for studying autophagy-related genes and pathways.
GEO	Gene Expression Omnibus	A public database for gene expression data and related information.
ARGs	Autophagy-related genes	Genes involved in the autophagy pathway.
MLKL	Mixed lineage kinase domain-like	A key effector in necroptosis, a type of programmed cell death.
RIPK3	Receptor-interacting serine-threonine kinase 3	A kinase essential for necroptosis and inflammation regulation.
TNFα	Tumor Necrosis Factor Alpha	A cytokine involved in systemic inflammation and immune system regulation.
TNFR	Tumor Necrosis Factor Receptor	A receptor for TNFα, mediating cell death or survival signals.
DRP1	Dynamin-Related Protein 1	A protein involved in mitochondrial fission and energy metabolism.
NLRP3	NOD-, LRR-, and Pyrin Domain-Containing Protein 3	A component of the inflammasome complex, crucial for immune response.

NF-kappa B pathway	Nuclear Factor Kappa B Pathway	A signaling pathway that regulates inflammation, immunity, and cell survival.
RNAs	Ribonucleic Acids	Molecules essential for coding, decoding, regulation, and expression of genes.
TAM M2	Tumor-associated macrophage M2	A subtype of macrophages associated with tumor progression and immune suppression.
GLUT inhibitors	Glucose Transporter Inhibitors	Compounds that inhibit glucose uptake in cells, used in metabolic therapy.
TPSAB1	Tryptase Alpha/Beta 1	A gene encoding a protease involved in inflammation and allergic responses.
TSPAN11	Tetraspanin 11	A member of the tetraspanin family, involved in cell adhesion and signaling.
CCDC92	Coiled-Coil Domain Containing Protein 92	A protein with roles in cellular structure or signaling.
KPNA2	Karyopherin Subunit Alpha 2	A nuclear import protein critical for transport of molecules into the nucleus.
NPM3	Nucleophosmin 3	A nucleolar protein associated with cell proliferation and stress response.
TWIST2	Twist-Related Protein 2	A transcription factor involved in mesenchymal transition and cancer progression.
H2AFZ	H2A Histone Family, Member Z	A variant of histone H2A involved in chromatin remodeling and gene regulation.
GAS1	Growth Arrest-Specific Protein 1	A protein that regulates cell growth and apoptosis.
CLEC3B	C-Type Lectin Domain Family 3, Member B	A protein potentially involved in coagulation and immune response.
TCGA	The Cancer Genome Atlas	A project to catalog genetic mutations responsible for cancer.
ROC	Receiver operating characteristic	A graphical representation to assess diagnostic test performance.
5-MPS	5-metabolic pathways	A compound with potential therapeutic applications.
ssGEA	Single-Sample Gene Set Enrichment Analysis	A computational method for functional analysis of gene expression data.
DDN-AS1	Dedicator of Cytokinesis 1 Antisense RNA 1	A long non-coding RNA with potential regulatory functions in cancer.
CNL	C6-ceramide nanoliposome	
Kif5b	Kinesin Family Member 5B	A motor protein involved in intracellular transport.
mTOR	Mechanistic Target of Rapamycin	A kinase involved in cell growth, metabolism, and survival.
TNKS1	Tankyrase 1	Poly(ADP-ribose) polymerases involved in telomere maintenance and Wnt signaling.
TNKS2	Tankyrase 2	Poly(ADP-ribose) polymerases involved in telomere maintenance and Wnt signaling.
UPR	Unfolded protein response	A cellular stress response related to endoplasmic reticulum stress.
CSCs	Cancer stem cells	A subpopulation of cancer cells with self-renewal and differentiation capacity.

GSEA	Gene Set Enrichment Analysis	A method to interpret gene expression data by focusing on biological pathways.
SRG score	Stress Response Gene Score	A metric indicating the activity of stress-response pathways.
ENE	Extranodal extension	Spread of cancer beyond lymph nodes into surrounding tissues.
DOI	Depth of invasion	A measure of how deeply cancer invades tissue layers.
AJCC	American Joint Committee on Cancer	Organization for cancer staging guidelines.
IFNG response	Interferon Gamma Response	A pathway involved in immune responses against infections and cancer.
INSL3	Insulin-Like 3	A hormone involved in testicular descent and reproductive biology.
FoxP3 CD4 Tregs	Forkhead Box P3 CD4+ Regulatory T Cells	Immune cells involved in suppressing immune responses and maintaining tolerance.
RBP4	Retinol-Binding Protein 4	A protein that transports vitamin A in the blood.
EGFR signaling pathway	Epidermal Growth Factor Receptor Signaling Pathway	A pathway critical for cell proliferation and survival, often deregulated in cancer.
MMP expression	Matrix Metalloproteinase Expression	Involves enzymes that degrade extracellular matrix, crucial for tissue remodeling and cancer.
CSF-1R-IL10 fusion protein	Colony-Stimulating Factor 1 Receptor Interleukin 10 Fusion Protein	A synthetic fusion protein designed for immunomodulatory therapy.
